# Eligible patients with heart failure prescribed vs. not prescribed vericiguat in the United States

**DOI:** 10.3389/fcvm.2025.1633435

**Published:** 2025-08-26

**Authors:** Stephen J. Greene, Catelyn R. Coyle, Lucy N. Hancock, Kathryn W. Tebbs, Sophie G. Barlow, Andra S. Stevenson, Engels N. Obi

**Affiliations:** ^1^ Duke Clinical Research Institute, Durham, NC, United States; ^2^ Division of Cardiology, Duke University School of Medicine, Durham, NC, United States; ^3^ Value and Implementation, Merck & Co., Inc., Rahway, NJ, United States; ^4^ Adelphi Real World, Bollington, United Kingdom

**Keywords:** vericiguat, heart failure, eligibility, United States, ejection fraction

## Abstract

**Aims:**

This study compared characteristics of patients with heart failure (HF) prescribed vericiguat vs. eligible patients with HF not prescribed vericiguat, and sought to identify factors associated with vericiguat use in real-world settings.

**Methods:**

We analysed 2022–2023 Adelphi HF cross-sectional survey from United States physicians and their adult patients. Patients prescribed vericiguat were compared with patients eligible for but not prescribed vericiguat. Vericiguat eligibility was defined as ≥1 prior HF hospitalization at any time, ejection fraction (EF) <45%, and no stage 5 chronic kidney disease or need for dialysis. Both cohorts were compared descriptively, and logistic regression used to identify factors associated with vericiguat non-use.

**Results:**

Overall, 93 physicians reported data on 228 patients with HF (mean age [SD]: 66.8 years [11.8], 65.7% male, 60.1% White), with 98 patients prescribed vericiguat and 130 patients eligible but not prescribed vericiguat. Patients eligible but not prescribed vericiguat had more comorbid hypertension (62.3% vs. 45.9%), hyperlipidemia (52.3% vs. 34.7%), and lower EF (mean [SD]: 34.7% [5.8%] vs. 41.7% [9.6%]), all *p* < 0.05. For every 1% increase in EF above 38%, odds of being prescribed vericiguat increased by 44% (Odds Ratio [CI]: 1.44 [1.28, 1.63]; *p* < 0.05).

**Conclusion:**

Among patients with HF in contemporary US clinical practice, patients prescribed vericiguat have distinct demographic and clinical profiles compared to eligible patients not prescribed vericiguat. Future research should confirm these findings and explore whether subgroups of eligible patients less likely to be prescribed vericiguat may benefit from targeted implementation initiatives.

## Introduction

Patients with worsening heart failure events (WHFE), defined as heart failure hospitalization (HFH) or use of outpatient intravenous diuretics, are at increased risk for downstream HFH and cardiovascular (CV) mortality (1). Vericiguat, a first-in-class soluble guanylate cyclase stimulator, received approval in the United States (US) in 2021 for the treatment of HF with reduced ejection fraction (HFrEF) following a WHFE based on findings from the VICTORIA clinical trial (2). Subsequently, the 2022 American Heart Association/American College of Cardiology (AHA/ACC) guidelines recommend vericiguat to reduce the risk of HFH and CV mortality following a WHFE, among patients with HFrEF receiving guideline-directed medical therapy (GDMT) (3).

However, despite proven benefits (2), utilization of vericiguat remains low in the US (4). Understanding factors associated with non-use of vericiguat among eligible patients in real-world clinical settings is important to inform targeted implementation strategies aimed at reducing the impact of HF-related morbidity and mortality in this population. This study compared the demographic and clinical characteristics of patients prescribed vericiguat, to eligible patients not prescribed vericiguat, and identify factors associated with vericiguat use in real-world settings.

## Methods

### Study design and data source

Data were collected from the Adelphi Real World HF Disease Specific Programme (DSP)™, a cross-sectional survey of physicians and their consulting patients, conducted in the US between August 2022 and February 2023. DSP methodology has been previously described and validated (5, 6).

Data collection adhered to the European Pharmaceutical Marketing Research Association guidelines and therefore ethics committee approval was not required.

### Study population and variables

Physicians completed an electronic patient record form (ePRF) for ≤10 consecutively consulting patients with HF and 1 additional ePRF for their next consulting patient with HF prescribed vericiguat. Patients eligible for inclusion in the DSP were ≥18 years of age, had a physician-confirmed diagnosis of HF, and were not participating in a clinical trial.

Patients were grouped into two cohorts: “patients eligible but not being prescribed vericiguat” had a history of ≥1 HFH at any time, a most recent EF <45% (based on the HFrEF definition in the VICTORIA trial) (2), and had no evidence of chronic kidney disease Stage 5 or need for dialysis. “Patients prescribed vericiguat” were prescribed vericiguat at the time of survey. Data collected included patient demographic and clinical characteristics, medical history, laboratory measures, and current HFrEF treatment.

### Statistical analysis

**Figure 1:**
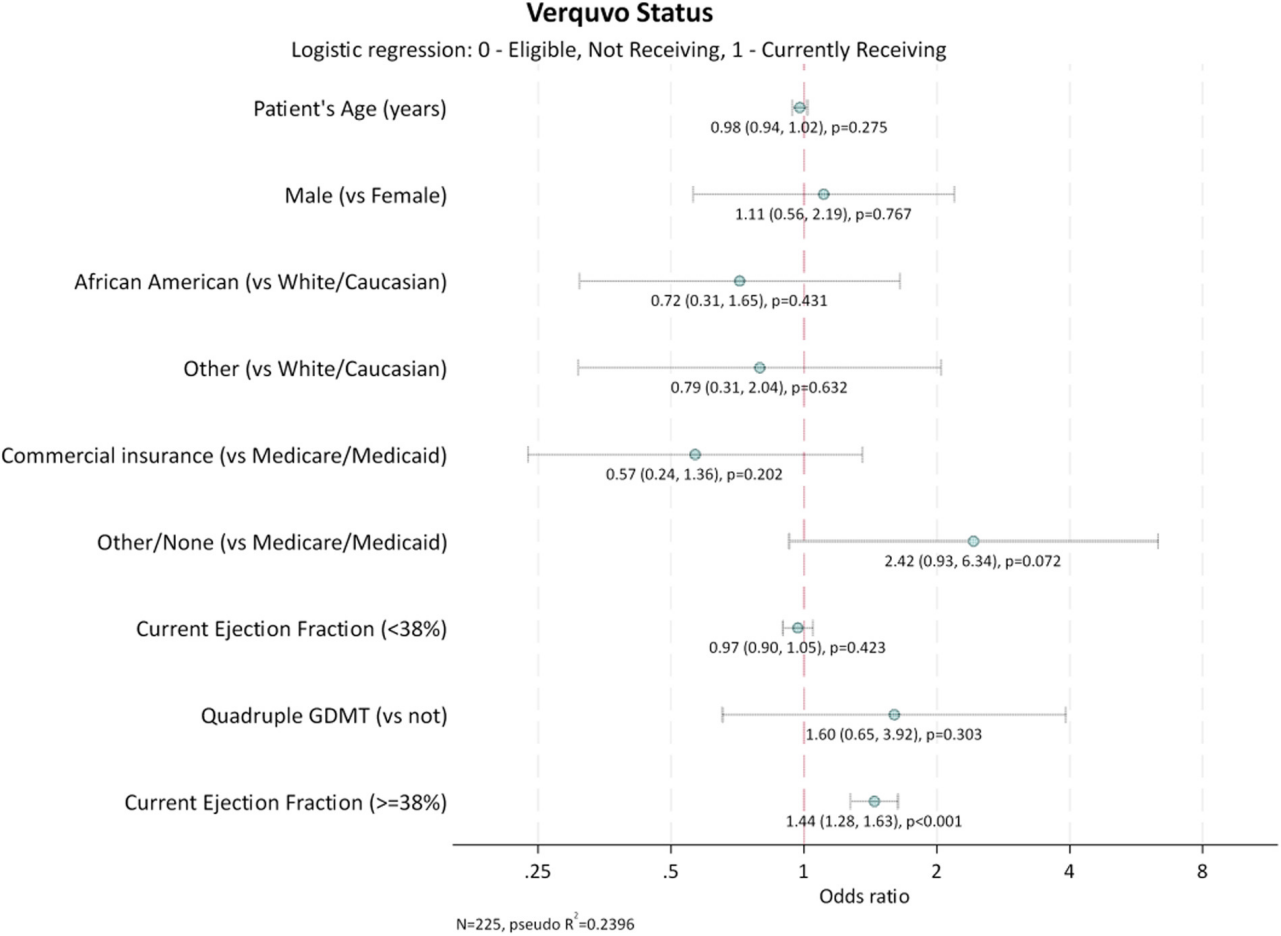
Factors associated with vericiguat use. GDMT, guideline-directed medical therapy. Quadruple GDMT was defined as use of angiotensin receptor neprilysin inhibitors (ARNi) or angiotensin-converting enzyme inhibitors (ACEi) or angiotensin II receptor blockers (ARB), and beta-blockers, and mineralocorticoid receptor antagonists (MRA), and sodium-glucose cotransporter-2 inhibitors (SGLT2i). Age and EF are reported as continuous variables. Sex, ethnicity, insurance status and receiving quadruple GDMT are reported as non-continuous variables. Interpretation: Odds ratio per 1% increase of ejection fraction.

Characteristics of patients who were prescribed vericiguat were compared with those of patients eligible but not prescribed vericiguat. Comparisons utilized Mann–Whitney tests for ordered categorical variables, *T*-test for continuous variables, and Fisher's Exact Test for nominal categorical variables. Additionally, a logistic regression was used to identify factors associated with vericiguat use (Figure 1). Ejection fraction linearity was assessed and there was found to be a non-linear relationship, so a linear spline with knot at the median value of EF (38%) was used within the model. Covariates included in the model are listed in the footnote to Figure 1.

All analyses were conducted using Stata 18 (StataCorp 2023. Stata Statistical Software: Release 18. College Station, TX: StataCorp LLC).

## Results

A total of 93 physicians (58 [61.7%] cardiologists and 36 [38.3%] primary care physicians) provided information for 228 patients with HF. This study cohort included 98 (43.0%) patients prescribed vericiguat, and 130 (57.0%) patients eligible but not prescribed vericiguat.

Most demographics, clinical characteristics, and treatment patterns were similar in the two groups (Table 1). However, patients prescribed vericiguat were more frequently from the Midwest (39.8%) and 44.6% of the patients eligible but not prescribed vericiguat were likely from the West (44.6%). Health insurance other than Medicare, Commercial or Medicaid insurance was more common among patients prescribed vericiguat than patients eligible but not prescribed vericiguat (14.3% vs. 4.6%, *p* = 0.016).

**Table 1:** Patient demographics, characteristics, and treatment patterns by prescription of vericiguat.

Variable	Overall (*n* = 228)	Eligible, not prescribed vericiguat (*n* = 130)^a^	Prescribed vericiguat (*n* = 98)^b^	*p*-value
Age				
Years, mean (SD)	66.8 (11.76)	67.4 (12.21)	66.0 (11.15)	0.383
Sex				
Male sex, *n* (%)	150 (65.79)	88 (67.69)	62 (63.27)	0.573
Ethnicity				
White, *n* (%)	137 (60.09)	76 (58.46)	61 (62.24)	0.587
Black, *n* (%)	44 (19.30)	28 (21.54)	16 (16.33)	0.397
Hispanic, *n* (%)	28 (12.28)	15 (11.54)	13 (13.27)	0.690
Other^c^, *n* (%)	22 (9.65)	12 (9.23)	10 (10.20)	0.824
US region				
West, *n* (%)	79 (34.65)	58 (44.62)	21 (21.43)	<0.001
Northeast, *n* (%)	67 (29.39)	39 (30.00)	28 (28.57)	
Midwest, *n* (%)	59 (25.88)	20 (15.38)	39 (39.80)	
South, *n* (%)	23 (10.09)	13 (10.00)	10 (10.20)	
Insurance status				
Medicare, *n* (%)	128 (56.14)	75 (57.69)	53 (54.08)	0.593
Commercial, *n* (%)	72 (31.58)	46 (35.38)	26 (26.53)	0.195
Medicaid, *n* (%)	11 (4.82)	4 (3.08)	7 (7.14)	0.213
Other^d^, *n* (%)	20 (8.77)	6 (4.62)	14 (14.29)	0.016
No insurance coverage	2 (0.88)	0 (0.00)	2 (2.04)	0.184
Top 5 comorbidities				
Hypertension, *n* (%)	126 (55.26)	81 (62.31)	45 (45.92)	0.016
Hyperlipidemia, *n* (%)	102 (44.74)	68 (52.31)	34 (34.69)	0.011
Diabetes, *n* (%)	73 (32.02)	48 (36.92)	25 (25.51)	0.085
Osteoarthritis, *n* (%)	37 (16.23)	25 (19.23)	12 (12.24)	0.204
Depression, *n* (%)	35 (15.35)	19 (14.62)	16 (16.33)	0.716
Time since HF diagnosis^e^				
Mean (SD) years	2.9 (2.87)	3.2 (3.19)	2.4 (2.23)	0.072
HF hospitalization^f^				
Never, *n* (%)	42 (20.00)	0 (0.00)^g^	42 (52.76)	<0.001
Within past 6 months, *n* (%)	22 (11.17)	18 (14.63)	4 (5.41)	
More than 6 months ago, *n* (%)	133 (65.51)	105 (85.37)	28 (37.84)	
Time since most recent hospitalization (days)				
Median (IQR)	90.5 (22.0, 220.5)	84.5 (17.0, 185.0)	201.0 (62.0, 242.0)	0.498
EF at time of visit				
Mean (SD)	37.71 (8.43)	34.69 (5.83)	41.71 (9.62)	<0.001
Current treatment				
Angiotensin converting enzyme inhibitor (ACEi), *n* (%)	66 (28.95)	53 (40.77)	13 (13.27)	<0.001
Angiotensin receptor blockers (ARB), *n* (%)	32 (14.04)	26 (20.00)	6 (6.12)	0.003
ACEi/ARB, *n* (%)	90 (39.47)	71 (54.62)	19 (19.39)	<0.001
Angiotensin receptor blocker + neprilysin inhibitor (ARNi), *n* (%)	94 (41.23)	62 (47.69)	32 (32.65)	0.030
Beta-blockers, *n* (%)	163 (71.49)	118 (90.77)	45 (45.92)	<0.001
Mineralocorticoid receptor antagonists (MRA), *n* (%)	68 (29.82)	37 (28.46)	31 (31.63)	0.662
Sodium-glucose cotransporter-2 inhibitors (SGLT2i), *n* (%)	62 (27.19)	37 (28.46)	25 (25.51)	0.654
Receiving triple therapy				
Yes, *n* (%)^h^	28 (12.28)	17 (13.08)	11 (11.22)	0.839
No, *n* (%)	200 (87.72)	113 (86.92)	87 (88.78)	
Receiving quadruple GDMT				
Yes, *n* (%)^i^	32 (14.04)	20 (15.38)	12 (12.24)	0.566
No, *n* (%)	196 (86.0)	110 (84.62)	86 (87.76)	

BMI, body mass index; EF, ejection fraction; eGFR, estimated glomerular filtration rate; GDMT, guideline-directed medical therapy; HF, heart failure; IQR, interquartile range; NT-proBNP, N-terminal pro–B-type natriuretic peptide; SD, standard deviation; US, United States. Data points which add to >100% are due to the question being multiple choice and some respondents selecting >1 option.

^a^ Eligibility criteria for “Eligible but not currently prescribed vericiguat:” Patients eligible but not being prescribed vericiguat at the time of survey had a history of ≥1 HFH at any time, an EF <45% at their last assessment, were part of the random sample, and had no evidence of chronic kidney disease Stage 5 or need for dialysis.

^b^ Eligibility criteria for “Currently prescribed vericiguat:” HF diagnosis and being prescribed vericiguat at the time of survey.

^c^ Comprised of Native American, Asian (Indian subcontinent), South-East Asian, Asian (other), Middle Eastern and Other not included in the list.

^d^ Comprised of Health Insurance Exchange Plan, Cobra (continuation coverage), Non-Medicare Retired Benefit and Tricare/Veterans Healthcare.

^e^ Overall, 175 patients had data stating how long it had been since they were diagnosed with HF, 104 among patients eligible but not prescribed vericiguat, and 71 among patients who were prescribed vericiguat.

^f^ Overall, 210 patients had data about previous HFH, 130 among patients eligible but not prescribed vericiguat, and 80 among patients who were prescribed vericiguat.

^g^ Inclusion criteria for patients eligible but not prescribed vericiguat included history of HFH. As a result, all patients in this group have ≥1 HFH in their medical record.

^h^ Triple therapy was defined as angiotensin receptor neprilysin inhibitors (ARNi) or angiotensin-converting enzyme inhibitors (ACEi) or angiotensin ii receptor blockers (ARB), and beta-blockers, and mineralocorticoid receptor antagonists (MRA). Note that patients receiving quadruple therapy which included the triple treatment defined here were excluded from these analyses.

^i^ Quadruple GDMT was defined as use of ARNi or ACEi or ARB, and beta-blockers, and MRA, and sodium-glucose cotransporter-2 inhibitors (SGLT2i).

Compared to patients prescribed vericiguat, those eligible but not prescribed vericiguat had a higher prevalence of hypertension (62.3% vs. 45.9%, *p* = 0.016), and hyperlipidemia (52.3% vs. 34.7%, *p* = 0.011), but lower ejection fraction [(EF) 34.7% vs. 41.7%, *p* < 0.001]. Compared to patients prescribed vericiguat, a higher proportion of patients eligible but not being prescribed vericiguat received at least 1 GDMT drug class, including beta-blockers (90.8% vs. 45.9%, *p* < 0.001), angiotensin receptor neprilysin inhibitors [(ARNi) 47.7% vs. 32.6%, *p* = 0.030], angiotensin-converting enzyme inhibitors [(ACEi) 40.8% vs. 13.3%, *p* < 0.001], and angiotensin II receptor blockers [(ARB) 20.0% vs. 6.1%, *p* = 0.003]. Only a small proportion of patients in both groups were receiving triple (overall: 12.2%) or quadruple (overall: 14.0%) GDMT.

### Factors associated with vericiguat prescription

For every 1% increase in EF at and above the median (≥38%), the odds of being prescribed vericiguat increased by 44% [odds ratio (CI): 1.44 (1.28, 1.63); *p* < 0.001], although among lower EF <38% there was no significant relationship. All other candidate covariates did not have a significant association with vericiguat use.

## Discussion

Although prior studies have described the clinical profile of patients eligible for vericiguat (7), the current study examined how eligible patients compare with patients actually prescribed vericiguat in US clinical practice. Moreover, the current study also identified factors independently associated with vericiguat use in the US, highlighting patient subgroups where targeted implementation efforts may be particularly needed.

Patients prescribed vericiguat were more often from the Midwest while patients eligible but not prescribed vericiguat were more often from the West, highlighting potential geographic differences in clinician practices. Additionally, it should be noted that, by definition, all eligible patients not receiving vericiguat in the current study had a history of HFHs and exhibited a lower mean EF, indicating substantial risk of downstream WHFEs. Non-prescription of vericiguat in this population can be viewed as a missed opportunity for further reducing residual clinical risk, with current HF guidelines recommending consideration of vericiguat following WHFE to reduce subsequent rates of cardiovascular death or HF hospitalization.

Overall, only 14% of patients in the study sample were prescribed quadruple GDMT. This is consistent with prior studies that have found low rates of quadruple GDMT use ranging from 0.8% to 15.3% among patients with HF (8, 9), which suggests potential treatment inertia. Prior data suggest that use of vericiguat may be particularly helpful in 2 key patient profiles with worsening HF: (1) patients already receiving standard GDMTs in order to lower their residual clinical risk, and (2) patients unable to tolerate or with contraindications to other GDMTs. In the current study, use of background ACEI/ARB/ARNI and beta-blocker therapy were significantly lower among patients prescribed vericiguat compared with patients eligible but not prescribed vericiguat. This could suggest preferential use of vericiguat in this second clinical profile of patients unable to tolerate various components of quadruple therapy. From the standpoint of tolerability, vericiguat has minimal to no effect on systolic blood pressure and kidney function. Likewise, vericiguat can be initiated with an eGFR as low as 15 ml/min/1.73 m^2^ (2, 10, 11). In combination, these features support the strong safety and tolerability profile of vericiguat among patient potentially ineligible or intolerant to other GDMTs. The analysis of factors associated with vericiguat use revealed increased odds of vericiguat use in patients with higher EF across the ranges of EF ≥38% to <45%. There was no relationship between EF and vericiguat prescription among patients with EF < 38%. Whether this relationship among patients with EF ≥38% reflects a true tendency among prescribers vs. a chance finding requires confirmation in future studies with large sample sizes. Likewise, although other candidate variables such as age, sex, race, and insurance status were not significantly associated with likelihood of vericiguat prescription in the current study, these results should be verified in larger samples of patients.

The current study has several limitations inherent to survey research, such as sample and recall bias and unobserved data (e.g., EF at the point of treatment initiation). Firstly, patients in the vericiguat prescription group were chosen based on their prescription status, whereas the comparator group of eligible patients not prescribed vericiguat were randomly chosen based on eligibility criteria. Hence, these data cannot be used to estimate the rate of vericiguat uptake in clinical practice. Further to this, the eligible but not prescribed vericiguat group used history of HF hospitalization at any time as a criterion for inclusion. This limitation is relevant as it must be considered that this group includes patients who may have been hospitalized long before this study and have since remained clinical stable on optimized treatment. Second, the sample size was modest and future studies with larger sample sizes are needed to confirm these findings. The regression analysis was limited by sample size, and we were unable to include all potential covariates that may be associated with the outcome. Non-significant covariates may be due to insufficient power to detect small differences between groups. Thirdly, data on the clinician's reasoning for prescribing vericiguat were not collected, and the role of clinician perception of safety and tolerability in driving vericiguat prescription remains speculative. This limitation is relevant when considering the observed inverse association between use of vericiguat and other GDMTs. For example, it remains unclear if clinicians perceived patients on quadruple medical therapy as already “optimally treated”, potentially prompting less use of vericiguat. Furthermore, the degree to which clinicians might consider the specific patient comorbidities when making prescription decisions is unclear (12). Likewise, the underlying clinical rationale for patients with lower EF being less likely to be prescribed vericiguat remains unknown. Future dedicated studies of physician perceptions and decision-making surrounding prescription of vericiguat and other GDMTs are needed.

## Conclusion

Overall, our findings provide real-world insights into the patient profile of patients with HF prescribed vericiguat in routine US clinical practice, and the factors independently associated with vericiguat prescription among eligible patients. Future research efforts should aim to confirm these associations and explore whether subgroups of eligible patients less likely to be prescribed vericiguat may benefit from targeted implementation initiatives.

## Data Availability

All data that support the findings of this study are the intellectual property of Adelphi Real World. All requests for access should be addressed directly to lucy.hargreaves@omc.com.
